# Global Trends in Adolescent Health Inequalities and Their Social Determinants: A Bibliometric and Scoping Review

**DOI:** 10.3390/healthcare14020141

**Published:** 2026-01-06

**Authors:** Yang Wu, Xiaojuan Zeng, Zihan Zhou, Shiyou Wu

**Affiliations:** 1 Department of Sociology, Jiangxi University of Finance and Economics, Nanchang 330032, China2202320884@stu.jxufe.edu.cn (X.Z.);; 2 School of Social Work, Arizona State University, 411 N. Central Ave., Suite 800, Phoenix, AZ 85004, USA

**Keywords:** adolescents, health inequalities, social determinants, bibliometrics

## Abstract

**Objective:** To conduct a scoping review of the global trends of adolescent health inequities and their social determinants from 2000 to 2024 and establish an evidence base for developing targeted intervention strategies. **Methods:** Guided by the rainbow model, we conducted a bibliometric analysis of 171 peer-reviewed articles related to adolescent health inequalities and their social determinants from the Web of Science Core Collection using CiteSpace 6.3.1 to summarize empirical evidence on how social determinants of health (SDOH) influence adolescents’ health behaviors (e.g., drinking) and health outcomes (e.g., overweight). **Results:** First, results showed a progressive increase in publications addressing social determinants of adolescent health from 2000 to 2024. Journals in public health and preventive medicine accounted for the highest proportion of articles, with the United States contributing the largest national share (21.05% of global output). Second, an analysis of keywords showed that previous studies mostly focused on the effects of socioeconomic status, family affluence on adolescent health (e.g., physical activity, mental health, and overweight). Third, inequalities in adolescent health were prevalent globally. Health behaviors (e.g., diet, oral health, and smoking) have received widespread attention and are influenced by socioeconomic status, family environment, and gender, whereas various indicators of adolescent health outcomes (e.g., obesity, mental health, and suicide) were highly correlated with family socioeconomic status. **Conclusions:** To reduce adolescent health disparities, it is important to deepen interdisciplinary research, consider the impact of emerging societal (e.g., digital environments) and environmental factors (e.g., climate change), and develop systematic and comprehensive intervention strategies that encompass the individual, family, school, community, and national levels.

## 1. Introduction

Health equity means that everyone has a fair opportunity to be as healthy as possible [1]. In order to achieve health equity, everyone must have access to the conditions and resources that positively affect health, which are often referred to as the social determinants of health (SDOH). SDOH refer to living and working environments and their impacts on health that are determined by social status and resources rather than by disease itself [2], including quality of education, public safety, access to health care, social support, residential isolation, etc. [3]. These SDOH often interact with each other to create complex systems that influence long-term health outcomes [4,5].

Evidence from the Web of Science (WOS) suggests systematic disparities in adolescent health (health-promoting/undermining behaviors and health outcomes) [6,7]. Such inequitable health disparities are caused by social determinants, particularly the stratification of health status by social status, also known as the “Social Gradient in Health” [8]. Even health disparities caused by lifestyles, such as smoking and drinking, are due to the social environment that disables adolescents from being in control of their lives, which ultimately forces individuals to make choices that are not conducive to health [9].

Adolescence is recognized as a period that “provides important opportunities for prevention and intervention to support the healthy growth and development of young people, promote future health and well-being in adulthood, and thereby support the health of the next generation” [10]. This is a period of significant physical, cognitive, emotional, and social change [11,12]. Adolescents are increasingly aware of social processes and are highly susceptible to influences in the social environment that can lead to positive or negative health outcomes and, ultimately, to inequalities as adolescents age and transition into adulthood [13,14]. Therefore, identifying the social determinants of health behaviors (risks) and health outcomes in adolescents is essential to advancing our understanding of developmental health trajectories and developing targeted interventions. As emphasized by Viner et al., social factors that influence adolescent health are present at the individual, family, community, and national levels [12].

Therefore, in order to understand how different dimensions of SDOH affect adolescents’ health, this scoping review systematically examined the existing evidence on the impact of SDOH on international adolescent health behaviors and health outcomes. First, based on the theoretical framework of the rainbow model, a framework for analyzing SDOH was constructed, and the WOS Core Collection database was used to search and organize the literature by SDOH domains of health (inequality), socioeconomic status (SES), health care access and quality, and social and community context. Second, CiteSpace software was used to analyze the relevant literature in terms of publication volume, major journal sources, study countries, and keywords. Finally, existing evidence on the role of SDOH in adolescent health across a wide range of health indicators (health behaviors versus health outcomes) was assessed and summarized. In general, this review aims to explore the global research trends and major social determinants of adolescent health inequalities from 2000 to 2024.

## 2. Materials and Methods

### 2.1. Data Source

This study integrates bibliometrics with scoping review methods. This hybrid design leverages bibliometrics (quantitative trend analysis via CiteSpace 6.3.R1 Basic, which was developed by Dr. Chaomei Chen in 2006) and scoping review elements (qualitative evidence synthesis guided by PRISMA-ScR). Bibliometrics addressed research volume, collaboration networks, and keyword evolution. Scoping methods synthesized empirical evidence on SDOH mechanisms, enabling dual analysis of trends and determinants.

Since the bibliometric nature of this study required consistent citation network data, only the WOS Core Collection was searched to ensure analytic comparability [15]. The WOS Core Collection, as a curated subset of journals, books, and proceedings with standardized citation indexing, was exclusively used to ensure analytic consistency. It was selected for this bibliometric scoping review focusing on research trends because of its standardized citation indexing, which optimizes bibliometric comparability. Although CiteSpace can also process Scopus data, Web of Science’s curated metadata deliver more consistent algorithm results—like burst detection and centrality metrics—without artifacts introduced by cross-platform normalization.

The search strategy was TS = ((adolescen* OR youth* OR teen*) AND (health inequit* OR health equit* OR health inequalit* OR health equalit* OR social gradient*) AND (social determinant* OR socioeconomic determinant* OR structural determinant*)).

Since 2000, there has been a steep increase in the number of published articles related to adolescent health inequality and its social determinants. Therefore, the time span was set from 1 January 2000 to 31 December 2024. The type of literature was “article”, and the language was “English”.

### 2.2. Theoretical Framework

**Figure 1:**
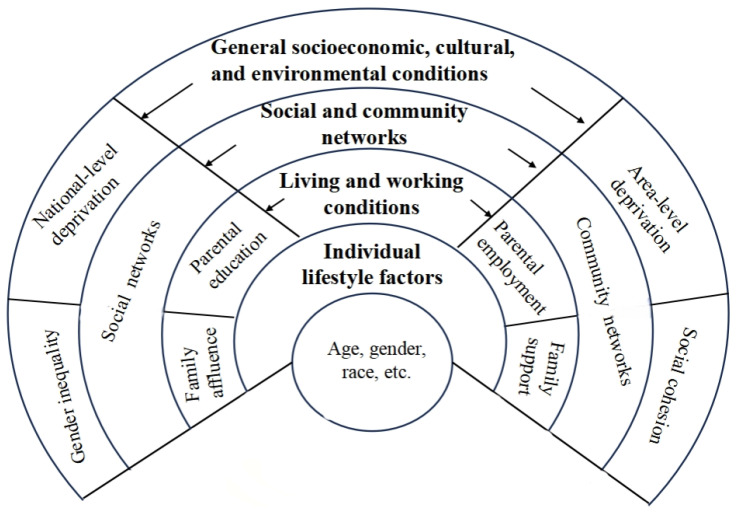
The Social Determinants of Health Model (adapted from Dahlgren and Whitehead’s rainbow model [16]). The model illustrates the layered structure of determinants, ranging from broad societal conditions to individual characteristics. Terminology and examples within each level are directly aligned with the analytical sections of this review.

In this study, a framework for modeling SDOH was constructed based on the Dahlgren and Whitehead model (often referred to as the “rainbow model”) [16]. This model assumes that different SDOH can function simultaneously and that there are often complex interactions between SDOH at the individual level, in the local context, and in society as a whole [16]. Considering the specifics of the finalized 171 papers, SDOH factors were classified into five groups: (1) general socioeconomic, cultural, and environmental conditions; (2) living and working conditions; (3) social networks; (4) community networks (particularly school networks); and (5) individual lifestyle factors (i.e., age, gender, and education). These factors were chosen due to stronger supporting evidence in the literature (Figure 1).

### 2.3. The Research Process

This scoping review followed the PRISMA Extension for Scoping Reviews (PRISMA-ScR) framework. Key steps included protocol development, systematic search, dual independent screening, and narrative synthesis of social determinant domains [17].

First, a total of 2613 articles were retrieved from the WOS Core Collection based on the identified topics and search terms. Two researchers used Zotero’s automated function to remove duplicates (82 articles), which were further verified by manual inspection. Then, the researchers independently performed an initial screening of the retrieved 2613 articles with regard to their titles, keywords, and abstracts, eliminating 1765 articles. Of the remaining 766 articles, the researchers screened their full texts based on the inclusion and exclusion criteria and extracted relevant data, including outcome variables (i.e., health behaviors and health outcomes), exposure variables (social determinants), country, and study population. In short, two researchers independently screened titles, keywords, and abstracts. The remaining records underwent a full-text review. Disagreements were resolved by a third researcher, resulting in the inclusion of 171 papers.

All bibliometric analyses and visualizations generated by CiteSpace (including trend analyses, journal/country distributions, and keyword burst detection) were based on the final corpus of the 171 included articles. While the initial search yielded 2613 records, this refined set represents the core literature that directly addresses the review question, ensuring that the identified networks and trends are specific to the field of adolescent health inequalities and social determinants. This focused corpus provides sufficient coherence and density for meaningful bibliometric mapping and trend analysis.

Studies were included if they met all of the following criteria: (1) the research topic was related to adolescent health inequality and its social determinants; (2) they were empirical studies; (3) they were published in English; (4) they were studies on adolescents (12–18 years old) in general; and (5) they were published between 1 January 2000 and 31 December 2024. Studies were excluded if they met any of the following criteria: (1) the research topic was not related to adolescent health inequalities or its determinants; (2) they were non-empirical studies; (3) they were not written in English; (4) the participants were younger than 12 or older than 18; (5) the data were surveyed prior to 2000; and (6) the study population belonged to a special group (e.g., people with AIDS, sexual minorities, or pregnant adolescents). Although excluding those vulnerable groups may have limited the analysis of extreme inequalities, it ensured generalizability to the broader adolescent population.

Second, researchers examined the identified 171 articles on international adolescent health inequities and their social determinants using CiteSpace 6.3.1 software, sorting them by the volume of publications, release dates, journals, major research countries, as well as detecting research hotspots from keyword frequency analysis. Finally, an analytical model of the SDOH for this study was developed based on the rainbow model to explore the social determinants of both health behaviors and health outcomes among adolescents worldwide and to find out the major determinants of adolescent health.

### 2.4. Research Variables

The predictors were the SDOH, as shown in Figure 1. The study outcomes included both health behaviors and health outcomes. Health behaviors broadly refer to a range of health-promoting (e.g., diet, physical activity, sleep duration) and health-damaging (e.g., drinking, smoking, marijuana use, sedentary behavior) behaviors. Health outcomes included perceived health indicators (e.g., health complaints and self-rated health), mental health (e.g., depression and anxiety), malnutrition, injuries, and communicable and noncommunicable diseases.

## 3. Results

### 3.1. Identification and Selection of Primary Literature

**Figure 2:**
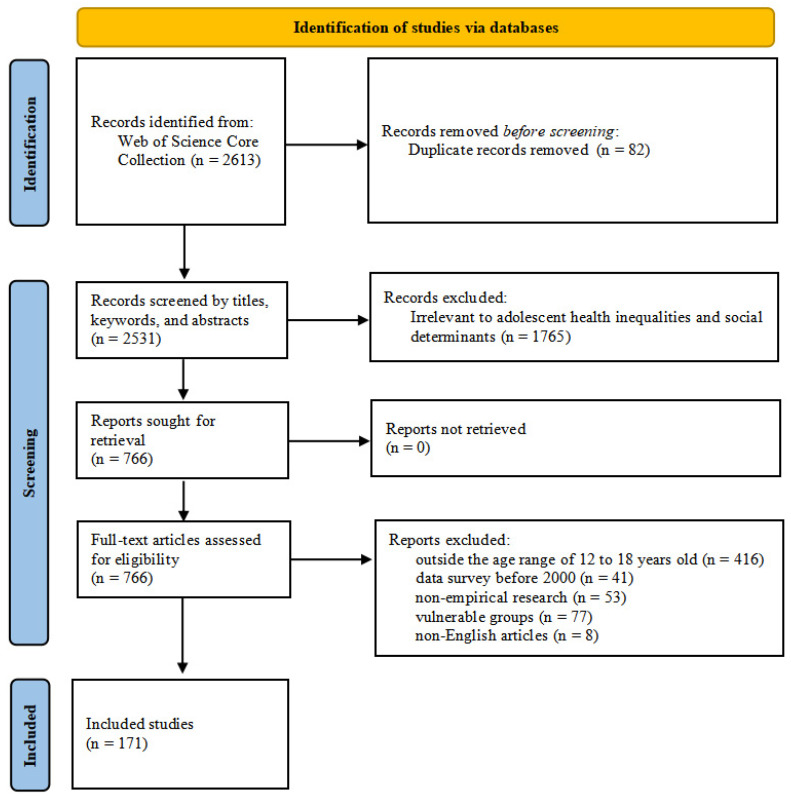
PRISMA-ScR flow diagram. (Included bibliographic data sourced exclusively from the WOS Core Collection).

Of the initial 2613 records, 766 studies were selected for full-text screening, of which 171 studies (articles) were finally selected. No additional records were identified that met the inclusion criteria after checking all the references of the included studies, resulting in a total of 171 studies being included in the analysis (see Figure 2). All studies were published between 2000 and 2024.

### 3.2. Quantitative Results of Citespace-Based Literature

#### 3.2.1. Trend Analysis of Literature Releases

**Figure 3:**
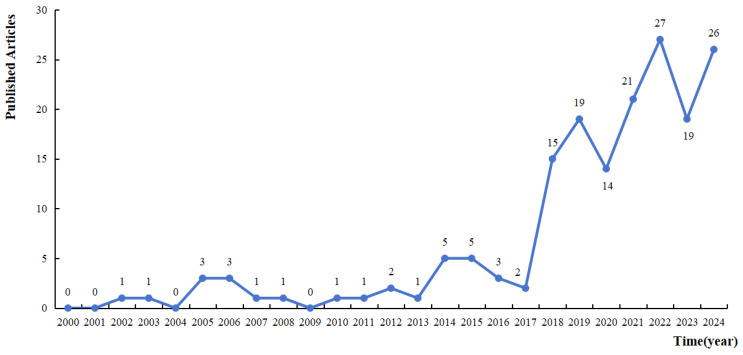
Trend analysis of the increase in the number of articles published in the study of social determinants of adolescent health, 2000–2024.

Trends in the number of publications in the field of research on adolescent health inequalities and their social determinants were analyzed through Microsoft Excel 2016 software. The results indicate a steady increasing trend in publications on adolescent health inequality (see Figure 3). In particular, the number of publications has increased significantly since 2018, indicating that the field is increasingly receiving attention from the academic community.

#### 3.2.2. Main Journal Sources

The international sample of social determinants of adolescent health was published in more than 90 journals, and the top 10 journals in terms of publications were mainly public health journals, with *BMC Public Health* in first place, followed by the *International Journal of Environmental Research and Public Health* and the *Journal of Epidemiology and Community Health*. This is shown in Table 1.

#### 3.2.3. Main Research Countries

Country attribution was determined by the institutional affiliation of the first author. For multi-country collaborations, the country of the corresponding author was prioritized. As shown in Table 2, the top five countries in the field of adolescent health inequities research in terms of publications over the past 24 years were the United States (n = 36), Canada (n = 29), the United Kingdom (n = 24), Australia (n = 17), and Germany (n = 14). Most of the research in this area was conducted in developed countries.

#### 3.2.4. Keyword Analysis

**Figure 4:**
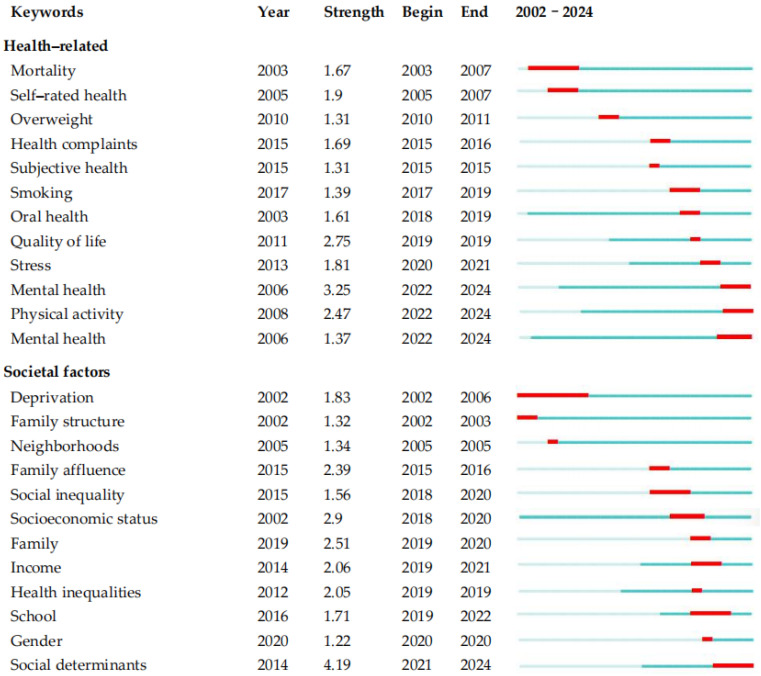
Top 12 keywords with the strongest citation bursts. (In the figure, light blue indicates the period that the keywords do not appear; dark blue indicates their active time. The red line highlights the burst period, when the keywords became a research hotspot. ‘Strength’ denotes the burst strength and is calculated based on a sudden increase in keyword frequency within a specific time frame).

Keywords were obtained from the title, abstract, and keyword sections of these articles, and those with relatively high frequency can be regarded as the research hotspots in the field to a certain extent. Meanwhile, according to the keyword attributes of the sample literature, the research hotspots of adolescent health inequality and its social determinants were categorized into two major groups: health-related and societal factors (see Figure 4).

For health-related keywords, “mortality” was the first to become a research hotspot, starting in 2003, and remained a research hotspot for 5 years, from 2003 to 2007. Since then, “self-rated health”, “overweight”, and keywords related to health behaviors (e.g., “smoking” and “physical activity”) have also begun to gain attention. The popularity of topics such as “anxiety”, “mental health”, and “physical activity” has continued into the present. In terms of strength (i.e., a sudden increase in the frequency of the keyword’s occurrence in a given time period), “mental health”, “quality of life”, and “physical activity” ranked as the top three, scoring 3.25, 2.75, and 2.47, respectively.

For keywords related to societal factors, “deprivation” was a hot topic in 2002–2006. “Neighborhoods”, “family affluence”, “income”, and “school” were also regarded as determinants of adolescent health. In terms of strength, “social determinants”, “socioeconomic status”, and “family” ranked as the top three, with 4.19, 2.9, and 2.51, respectively.

### 3.3. Evidence of Social Determinants of Health Behaviors Affecting Adolescents

The evidence of SDOH influencing adolescent health behaviors and health outcomes was summarized based on the rainbow model, from the individual level, family level, and social and community level to the general socioeconomic level. Of the 171 articles included in the literature, 81 were related to adolescent health behaviors, and according to these 81 articles, the social determinants all positively or negatively influenced (either promoted or undermined) health behaviors (see Table 3, Table 4, Table 5 and Table 6). The main adolescent health-promoting behaviors were physical activity, healthy diet, healthy oral habits (i.e., brushing teeth frequently and eating fewer sweets), sleep status, use of sexual and reproductive health services, use of health care or mental health services, having a good vaccination status, and having good health literacy and health perceptions [18,19]. Behaviors that impair the health of youth include alcohol and intoxication, tobacco use, marijuana use, bullying and violence, and unhealthy screen time habits [20].

#### 3.3.1. Individual Lifestyle Factors

Individual-level factors include age, gender, level of education (academic achievement), and ethnicity. Age, gender, and number of siblings were some of the factors that influenced whether an adolescent received good health care [21] (see Table 3). Of these, gender was the strongest predictor of health behaviors among adolescents [22]. Boys had significantly higher rates of smoking and breakfast consumption, higher levels of physical activity [23,24], but a lower frequency of tooth brushing compared to girls [25]. Boys were also more likely to avoid discussing menstruation-related topics. Educational level was also an important predictor, with adolescents with poor academic performance being more likely to engage in smoking, alcohol consumption, and marijuana use [26]. Additionally, ethnicity influenced adolescent health behaviors. Racial and ethnic minority adolescents, while being more likely to experience major depression (MDE), were less likely to use mental health (MH) services [27]. African American adolescents had higher levels of physical activity [28,29] and fewer sedentary behaviors [28] than other races, and boys from ethnic minorities smoked cigarettes and used alcohol at higher rates than non-ethnic minorities [30].

**Table 3:** Analysis of social determinants of inequalities in health behaviors: individual level.

Specific Influencing Factors	Health Behavior	No. of Studies
Age	Health service utilization (+): 1	2
	Cigarette smoking (+): 1	
Gender (for males)	Oral health behavior (−): 5 Health literacy (+): 3 Health service utilization (+): 2 Healthy diet (+): 2 Sports activity (+): 2	15
	Cigarette smoking (+): 1	
Race/ethnicity (for White)	Physical activity (−): 2 Healthy diet (+): 2 Vaccinations (+): 1	9
	Tobacco, alcohol, marijuana use (−): 1/(+):2 Sedentary behavior (+): 1	
Educational level	Healthy diet (+): 3 Oral health behavior (+): 2 Sporting activity (+): 2 Sleep condition (+): 1	13
	Smoking and drinking (−): 4 Unhealthy screen time habits (−): 1	
Psychological asset	Healthy diet, oral health behaviors, physical activity, sleep status (+): 2	3
	Smoking and drinking (−): 1	

(+ means positive effect, − means negative effect).

#### 3.3.2. Living and Working Conditions

In this analysis, “family affluence” (often measured by material assets or parental income) is treated as a specific component of family SES to differentiate its impact on adolescent health behaviors from that of other SES-related factors (i.e., parental education and occupation). The residential and living conditions of adolescents mainly refer to indicators of family SES, including family affluence, parental education, and parental occupation. There was a significant relationship between family SES and various adolescent health behaviors.

Specifically, in terms of family income, adolescents from more affluent families had increased odds of hazardous drinking [31] and intake of sugar-sweetened beverages [32], but at the same time, they were more likely to eat healthier [33] and have better oral health [26]. In contrast, adolescents from poorer families were most likely to have long screen use time [34], low breakfast frequency [35], low vaccination prevalence [36], sedentary behaviors [34], and physical fighting [37] (see Table 4).

**Table 4:** Analysis of social determinants of inequalities in health behaviors: family level.

Specific Influencing Factors	Health Behavior	No. of Studies
Family affluence	Sporting activity (+): 12 Healthy diet (+): 11 Health service utilization (+): 4 Oral health behavior (+): 3 Vaccinations (+): 2 Health literacy (+): 2 Sleep condition (+): 1	50
	Drinking (−): 6 Smoking (−): 5 Unhealthy screen time habits (−): 3 Physical fighting (−): 1	
Parents’ employment status and level of education	Healthy diet, oral health behaviors (+): 5 Vaccinations (+): 2 Health literacy (+): 1	10
	Drinking (−): 2	
Family support	Oral health behavior (+): 1	7
	Unhealthy screen time habits (−): 4 Cigarette smoking (−): 2	

(+ means positive effect, − means negative effect).

In terms of parental education level, adolescents with less-educated parents had lower vaccination rates [38] and physical activity levels [28,33] and higher odds of sugary drink intake [39]. In addition, parents’ occupation and employment status could negatively affect adolescent health [40]. For example, full-time parental employment and low companionship were associated with problematic media use in adolescents [41].

In terms of home environment, adolescents with poorer home environments also had lower utilization of health service interventions [42] and a higher risk of passive smoking [43], and they were more likely to develop addictive social media use [44]. In contrast, a good home environment and direct communication between family members could help adolescents reduce their intake of unhealthy foods [33] and increase their sports participation [26].

#### 3.3.3. Social and Community Networks

Social environment refers primarily to school-level SES. As shown in Table 5, the lower the school’s SES and the poorer the environment (e.g., public school or poor sanitation), the greater the odds that adolescents were exposed to unhealthy diets and alcohol consumption in school [45], and the poorer their oral health (e.g., low frequency of tooth brushing) [46,47]. Schooling is also a strong predictor of adolescents’ use of health facilities to access sexual and reproductive health information and services [48,49,50] and the development of health literacy [28,51,52]. Peers are also an important part of the social environment, and peer support can directly influence the amount of time adolescents spend in physical activity [28,29,53]. In addition, adolescents with lower peer status have lower oral health–related quality of life [54].

**Table 5:** Analysis of social determinants of inequalities in health behaviors: social and community-level.

Specific Influencing Factors	Health Behavior	No. of Studies
Community or neighborhood social capital	Healthy diet, oral health behaviors (+): 6 Sporting activity (+): 3	15
	Smoking and drinking (−): 5 Violence (−): 1	
School social capital	Healthy diet, oral health behaviors (+): 4 Sporting activity (+): 3 Vaccinations (+): 3 Health literacy (+): 2	13
	Smoking and drinking (−): 1	
Peer social capital	Healthy diet, oral health behaviors (+): 2 Sporting activity (+): 2	6
	Smoking and drinking (−): 1 Unhealthy screen time habits (−): 1	

(+ means positive effect, − means negative effect).

Community SES was correlated with adolescent physical activity facility utilization [55], injuries [56], self-rated health [57], and liquor store density [58]. For example, poorer neighborhoods have a greater number of fast food restaurants and fewer indoor facilities, and adolescents from poorer neighborhoods were more likely to be overweight [59].

#### 3.3.4. General Socioeconomic, Cultural, and Environmental Conditions

General socioeconomic, cultural, and environmental conditions include social deprivation at the national level (e.g., Gini coefficient and national income levels), at the regional level (area of residence and rural vs. urban), and cultural factors (see Table 6). The higher the income inequality at the national level, the more health-damaging behaviors—such as smoking and drinking [60,61,62], pathological gaming [63], problematic social media use [64], low levels of physical activity [65], and lower vaccination rates [66,67]—adolescents are likely to engage in.

**Table 6:** Analysis of social determinants of inequalities in health behaviors: general socioeconomic level.

Specific Influencing Factors	Health Behavior	No. of Studies
Social income inequality (e.g., Gini coefficient)	Sporting activity (−): 1	6
	Unhealthy screen time habits (+): 2 Bullying and violence (+): 1 Drinking (+): 1 Smoking (−): 1	
Area of residence	Healthy diet, oral health behaviors (+): 5 Vaccinations (+): 2 Health service utilization (−): 1	13
	Drinking and smoking, violent activities (−): 5	
Social mobility	Smoking (−): 1	1
Gender inequality	Violence (+): 1	1
National recommendations	Healthy diet, oral health behaviors (+): 1	1
COVID-19	Health service utilization (−): 1	2
	Unhealthy screen time habits, unhealthy diet (+): 1	
Social Vulnerability Index	Sleep condition (−): 1 Health service utilization (−): 3	4

(+ means positive effect, − means negative effect).

At the regional level, the higher the poverty rate, the poorer the oral health status of adolescents in that region, and socioeconomic factors at the regional level were associated with dental visits and frequency of tooth brushing [68]. Adolescents in rural areas or in socially disadvantaged situations also had lower utilization of sexual and reproductive health services [69] and mental health services [70,71,72]. In addition, more girls in rural areas were informed of serious menstrual contraindications [73], and they were far less likely to use sanitary measures during menstruation than in urban areas [74]. At the cultural level, there is a positive correlation between cultural capital and healthy food intake. For example, when adolescents engaged in elite cultural practices, healthy food intake increased [33].

It is important to note that all five groups of SDOH had an impact on the following health behaviors: physical activity, healthy diet, oral health behaviors, and tobacco and alcohol use. This suggests that inequalities in adolescent health behaviors are a hot area of research.

### 3.4. Social Determinants of Adolescent Health Outcomes

Of the 171 articles included in the literature, 121 were related to adolescent health outcomes, and the health outcome indicators covered adolescents’ self-reported health status, health complaints, injuries sustained, chronic mental health problems, depressed mood, overweight/obesity, thinness, developmental delays, and suicidality [75].

#### 3.4.1. Individual Lifestyle Factors

Age and loneliness were common correlates of overweight/obesity and suicidal thoughts in adolescents [72,76] (see Table 7). Gender was also associated with mental health problems in adolescents. Compared to girls, boys have significantly higher life satisfaction [77,78] and probability of perceived good health [79], as well as a lower likelihood of cardio-metabolic dysfunction [80] and health complaint rates [81] but worse oral health status [47].

The correlation between individual SES, as measured by academic achievement, and adolescent health outcomes (e.g., overweight, self-rated health status, and number of health complaints) was also very strong [82]. With poor academic achievement, adolescents were more likely to exhibit depressive symptoms and attempt suicide [83]. High early academic achievement was associated with lower youth homicide rates [84]. Individuals with lower educational attainment and Black adolescents had higher odds of developing chronic noncommunicable diseases across most risk factors [85].

In terms of individual past experiences, adverse childhood experiences were associated with poorer health [86], and cumulative socioeconomic disadvantage in adolescence was associated with poor oral health [87]. Increased experiences of religious discrimination were associated with increased emotional problems and decreased sleep duration [88]. Significant differences also existed between adolescents of different ethnicities in terms of their cardiovascular health [89] or other health indicators [47,90,91]. For example, those of South Asian or African descent had a higher risk of diabetes than White Europeans [92]; Filipino, Japanese/Korean, Southeast Asian, and South Asian adolescents were more likely to be obese than Chinese adolescents [93]. White athletes were more likely to report concussions than Black athletes [94], Black students were at higher risk for chronic diseases, and young people from the Caribbean, of mixed-race, or with other racial backgrounds had a higher prevalence of mental health problems [95].

#### 3.4.2. Living and Working Conditions

As shown in Table 8, adolescents with lower SES had poorer periodontal health [96] and were also more likely to be overweight [79,97,98,99,100], have physical and mental health problems [101,102], have high levels of perceived stress, and have low life satisfaction [77,103]. In contrast, adolescents with high SES and family capital were more likely to have high life satisfaction [104]. The status of specific indicators of family SES is shown below.

With respect to family income, in almost all countries, adolescents in low-income families tended to experience a poorer quality of life [105] and report more violent behavior [37,106], have poor oral hygiene [107,108,109], and they were also more likely to have mental health problems [97,103,110,111]. Furthermore, low household incomes also contributed to more mental health problems in conjunction with perceived economic well-being disadvantages at the national level [112,113].

In terms of parental education level, adolescents from families with higher educational backgrounds had a better quality of life in terms of physical health, mental health, and oral health [47,108,114], and a lower prevalence of asthma [91,115] and probability of obesity [93,98,99,100]. In contrast, adolescents with less educated parents had a higher risk of hospitalization for violence, self-harm, and substance abuse [116,117]. In terms of inter-generational educational level, adolescents with downward-intergenerational education had much poorer self-assessed health compared to their peers, with consistently higher levels of inter-generational education [118].

Adolescents from poorer family environments were also likely to have poorer self-rated health [78,119,120] and freedom from peer violence [121], and they tended to lack family support, communication, and cohesion. Health inequalities for adolescents from lower family economic backgrounds could also be somewhat mitigated if family cohesion was high [122].

#### 3.4.3. Social and Community Networks

As shown in Table 9, school-level SES greatly influences the quality of oral health [107]. Poorer school environments were associated with a higher prevalence of periodontal unhealthiness in adolescents [47,108,123]. School type is also an important predictor of mental health problems in adolescents [22]. If adolescents grew up in a country with an above-average size of the private pre-school sector, they tended to have less socioeconomic inequality in terms of multiple psychological burdens [124]. Inequalities in adolescent health complaints were smaller in countries with more stratified education systems [125].

Community SES is also associated with inequalities in adolescent health outcomes. Higher community SES was associated with a lower likelihood of long-term conditions and being overweight [126], fewer weekly health complaints, and better self-rated health [101].

#### 3.4.4. General Socioeconomic, Cultural, and Environmental Conditions

As shown in Table 10, country-level economic development and income inequality factors are recognized as key determinants of adolescent health outcomes [14], with large socioeconomic gradients across different health indicators in adolescents [12]. For example, the higher the country’s income inequality (Gini coefficient), the higher the prevalence of adolescent obesity [127], physical and mental health complaints [124], depression scores [128], oral problems [129], chances of bullying victimization [130], suicidality [131], and mortality rates [132]. Notably, scholars have also found that closure (social mobility) during the COVID-19 epidemic was associated with a higher prevalence of depressive symptoms in adolescents [133,134,135]. The level of social deprivation at the district level is mainly measured by the area of residence (upper-middle-class areas, migrant/resettlement areas, and urban slum areas). Adolescents in deprived areas had poorer oral hygiene [136,137] and poorer quality of life [78] as well as higher cardio-metabolic risks [138] and cancer incidence [139].

Cultural factors mainly refer to the degree of gender equality and social cohesion. Boys in countries with higher levels of gender inequality had higher levels of fighting and assault, while physical injuries were more common in countries with lower levels of gender inequality [140]. Adolescents living in more socially cohesive environments had higher levels of physical and mental health, higher levels of self-efficacy [141], and better academic performance [142].

## 4. Discussion

This scoping review presents research findings from the past 24 years (2000–2024) that are related to the social determinants of adolescent health and explores research hotspots as well as important determinants of adolescent health behaviors and health outcomes to provide insights into practice.

### 4.1. Quantitative Analysis of the Literature

The steady increase in publications from 2000 to 2024 highlights growing academic concern regarding adolescent health. This trend likely reflects two realities: adolescents are confronting multifaceted health crises, and identifying pathways to sustain their lifelong health and quality of life has become a global research priority [143]. In the 21st century, health has been emphasized more than ever before, and at the Millennium Summit of the United Nations (UN) held in September 2000, 189 member states signed the United Nations Millennium Declaration, which fully demonstrates that health is a comprehensive goal that can only be achieved by improving the social environment [144]. Recent studies confirm COVID-19 exacerbated mental health inequalities, aligning with our 2024 trend analysis [140].

Regarding major journal sources, *BMC Public Health* leads in the number of publications. This journal emphasizes interdisciplinary work across disease epidemiology, social determinants of health, and health policy. Closely following is the *International Journal of Environmental Research and Public Health*, which is dedicated to health promotion and disease prevention, fostering cross-disciplinary collaboration to advance population well-being.

In terms of national distribution, the United States is committed to the systematic interpretation of the SDOH. This focus is reflected in key policy frameworks. The Healthy People 2020 initiative (2010) included SDOH among its 42 topic areas and added the Youth Health Initiative [145]. Its successor, Healthy People 2030 (2020), further highlights disparities between actual and optimal health status [146]. The U.S. has captured changes in societal health issues and led the world in the iterative development of related research.

Keyword analysis revealed “mental health”, “quality of life”, and “physical activity” as the most prominent health-related hotspots. This may be due to their far-reaching and interrelated effects on adolescents’ health, with mental health problems being exacerbated by quality of life, which is closely related to physical activity [147,148]. The main research hotspots in SDOH are “social determinants” and “family”. Extensive evidence confirms that lower SES is associated with a higher incidence of health problems [110,149]. There is a link between household SES and health behaviors (e.g., diet and physical activity), with lower household SES generally reporting poorer eating habits [150,151] and lower levels of physical activity [152]. In addition, adolescents with lower family SES perform relatively poorly in areas such as perceptions of well-being and are prone to a form of “double disadvantage” in their physical and mental health [153].

### 4.2. Key Social Determinants of Health Behaviors

SDOH at all levels influence health behaviors, particularly healthy diet, oral health behaviors, and tobacco and alcohol use.

The social determinants of inequalities in dietary behaviors are mainly SES, family environment, social environment, and gender at the national level. First, countries with lower SES may be less endowed with material (e.g., increased food budgets and access to health-promoting goods and services) [154] or psychosocial resources (e.g., nutrition knowledge, cooking skills, and positive attitudes toward healthy eating) [155,156]. For example, evidence from Scandinavia’s school nutrition programs [33] demonstrates replicable models for LMICs. Mobile-health interventions in Brazil improved vaccine access [66]. Second, healthy eating behaviors are shaped by the family environment, which includes not only the food supply but also the eating behaviors of parents [157]. Parents in disadvantaged circumstances often discuss healthy eating less frequently and express lower levels of relevant concern [158]. Third, the sociocultural environment and peers also influence adolescents’ food consumption and eating habits. Focusing on the establishment of healthy eating behavior patterns among adolescents’ friend groups can help to promote healthy lifestyles among young people [159]. Fourth, boys report higher breakfast frequency than girls. This disparity may be driven by girls skipping breakfast for weight control or being influenced by maternal attitudes and behaviors [160] or by media portrayals of ideal body types [161].

The most important predictor of oral health behavior is family SES. Adolescents’ frequency of tooth brushing is influenced by family SES, such as the level of parental education, as well-educated parents are generally more concerned about their children’s oral health [162]. In general, a higher level of education leads to more opportunities to develop healthier habits, use health services, and benefit from health promotion activities [163].

Gender and cultural environment are the main factors influencing adolescents’ smoking and drinking. Smoking prevalence is higher among boys than girls, which may be influenced by gender role socialization (i.e., boys and girls are perceived or presented as more masculine or feminine because of their biological sex) [164]. In many countries, smoking is considered part of the social construction of masculinity.

### 4.3. Key Social Determinants of Health Outcomes

Among all levels of social determinants, family socioeconomic status (SES) shows the strongest correlation with key adolescent health outcomes, including obesity, mental health problems, and suicidal behavior. Adolescents with lower family SES have higher rates of personal psychological burden, multiple mental disorders, and poorer quality of life compared to their peers [165]. Because of unequal or lower family income, it is difficult for adolescents to access good housing, schools, and health care resources, which in turn reduces adolescents’ quality of life and well-being. Existing support programs for low-income families—such as TANF, the Children’s Health Insurance Program, SNAP, and school lunch programs—typically have stringent income eligibility thresholds. Many low-income families either do not qualify for assistance or receive only limited assistance [113].

An unfavorable home economic environment can also lead to an increased risk of suicidal and self-injurious behaviors among adolescents. First, adolescents in unfavorable situations in the home may be vulnerable to many stressors and more prone to have mental health problems [166]. Second, low SES is often linked to poor family functioning and parental mental or physical health issues, which can arise from unemployment, divorce, or separation [167]. These challenges, in turn, negatively affect parenting quality. Third, adolescents’ perception of their family’s low social class can diminish self-esteem, foster feelings of loneliness and depression, and thereby elevate the risk of suicidal thoughts and self-harm [168]. In contrast, the more family health assets an adolescent has, the lower the risk of poor health indicators [169].

It is important to note that SES-based health disparities among adolescents have persisted or even widened. Moreover, family SES alone is insufficient to fully protect adolescents from health-risk behaviors or poor self-reported health [170]. Instead, multidimensional health assets—encompassing factors at the individual, family, school, community, and policy levels—are more effective in shielding adolescents from risky behaviors and poor health while promoting positive outcomes [171].

## 5. Conclusions

This bibliometric analysis of 171 studies (2000–2024) reveals the following key findings: (1) The United States has made the largest contribution to research on adolescent health inequalities and their social determinants. (2) Health behaviors (e.g., diet, smoking, and oral health) have received extensive attention and have been demonstrated to be influenced by SES, family environment, and gender, respectively. (3) Family SES is the strongest predictor of adolescent health inequalities (e.g., mental health and obesity). (4) Post-2020 studies have highlighted digital inequality (e.g., problematic social media use) as a novel amplifier of mental health disparities.

The social determinants of adolescent health behaviors and health outcomes are complex and diverse. Based on summarizing relevant international research, this study proposes the following recommendations:

First, interdisciplinary research is needed. Given that adolescent health is influenced by multidimensional SDOH, future research should further strengthen interdisciplinary collaboration and encourage longitudinal studies and large-scale survey projects to better understand changes in adolescent health behaviors and health outcomes over time and their dynamic relationship with the social environment.

Second, the impact of emerging social and environmental factors should be taken into account. Future research should explore the impact of emerging social phenomena (e.g., social media use, digital environments, and climate change) on adolescent health behaviors and health outcomes, and how these factors interact with traditional social determinants. It should also examine the specific pathways by which cultural and social factors, such as gender inequality and social cohesion, influence adolescent health.

Third, integrated interventions should be prioritized. To disrupt pathways linking SDOH to adolescent health disparities, interventions must integrate multiple levels:

(a) Economic and educational leverage. For example, Bangladesh’s Female Secondary School Stipend Program (FSSSP) provides cash grants to rural girls for secondary education, raising the level of education of mothers, which in turn raises the rate of full immunization of their children [172]. (b) Policy and health care synergy. For example, Australia implemented a youth tobacco tax and e-cigarette ban, imported over-the-counter e-cigarettes, and increased smoking cessation clinics in low-income neighborhoods [173]. (c) Community-level innovation. For example, mobile health clinics in the United States provide care for some of the most vulnerable populations, such as adolescent health screenings [174]. These examples underscore that standalone programs are insufficient; systemic change requires coordinated action across individual, familial, community, and policy domains.

Finally, we recommend that integrated interventions prioritize the following concurrent actions: individual agency (e.g., health literacy), family support (e.g., parenting programs), community resources (e.g., mobile clinics), and policy reform (e.g., equitable taxation).

While the nature and source of health inequalities differ significantly from high-income countries, the second and fourth recommendations are particularly pertinent to low- and middle-income countries like China, as it is undergoing significant changes in social and environmental factors, while evidence on SDOH in adolescent health disparities is relatively limited.

## 6. Limitations of the Study

This scoping review has significant value in dissecting adolescent health inequalities and their social determinants, but some limitations remain. First, the latest included data were collected in 2022, with publications processed through 2024. This creates a lag in capturing post-pandemic societal shifts (e.g., digital inequality exacerbation and mental health impacts of prolonged isolation) that reshape adolescent health disparities. Second, country-specific differences have been ignored, and discussing the situation in developed and developing countries together may obscure some key issues. There are differences in the level of development, social structure, allocation of health resources, and cultural background across countries, all of which have an impact on adolescent health inequalities. More studies are needed to explore the differences between developed and developing countries. Third, while we identified key social determinants (e.g., family SES, gender, and community capital), our analysis did not deeply investigate how these factors dynamically intersect and amplify one another. This limits understanding of complex cascading effects across the rainbow model’s layers.

## Figures and Tables

**Table 1:** Top 10 journals with the strongest citation bursts.

Rank	Periodicals	No. of Documents/Article
1	*BMC Public Health*	12
2	*International Journal of Environmental Research and Public Health*	10
3	*Journal of Epidemiology and Community Health*	8
4	*Public Library of Science One*	7
5	*Social Science and Medicine—Population Health*	5
6	*Brazilian Oral Research*	5
7	*Social Science and Medicine*	4
8	*BMJ Open*	3
9	*Community Dentistry and Oral Epidemiology*	3
10	*European Journal of Public Health*	3

**Table 2:** Top 10 countries with the strongest citation bursts.

Rank	Periodicals	No. of Documents/Article	Percentage/%
1	USA	36	21.05
2	Canada	29	16.96
3	England	24	14.04
4	Australia	17	9.94
5	Germany	14	8.19
6	Brazil	12	7.02
7	Spain	11	6.43
8	Finland	10	5.85
9	Netherlands	10	5.85
10	Sweden	9	5.26

**Table 7:** Analysis of social determinants of inequalities in health outcomes: individual level.

Specific Influencing Factors	Health Behavior	No. of Studies
Age	Health (+): 1 Oral health (−): 1	6
	Mental health disorders (+): 1 Suicide rate (+): 1 Self-assessed health status (−): 1 Sickness (+): 1	
Gender (for males)	Self-assessed health status (−): 2/(+): 3 Oral health (−): 4 Life satisfaction (+): 2 Health-related quality of life (+): 1	16
	Self-reported symptoms of depression (+): 1 Physical injury (+): 1 Suicide rate (+): 1 Sexual (−): 1	
Race/ethnicity (for White)	Self-assessed health status (+): 3 Oral health (+): 3 Sleep health (+): 2	16
	Sickness (−): 3 Clinical depression (−): 1 Obese (−): 1 Sexual (−): 1 Physical injury (−): 1 Likelihood of HIV infection (−): 1	
Education level	Self-assessed health status (+): 7 Oral health (+): 1	13
	Sickness (−): 2 Clinical depression (+): 1 Substance use disorders (−): 1 Homicide mortality rate (−): 1	
Being bullied	Life satisfaction, physical and mental health (−): 1	1

(+ means positive effect, − means negative effect).

**Table 8:** Analysis of social determinants of inequalities in health outcomes: family level.

Specific Influencing Factors	Health Behavior	No. of Studies
Family affluence	Self-assessed health status (+): 14 Oral health (+): 12 Life satisfaction (+): 8 Sleep health (+): 1	78
	Physical and mental health complaints (−): 21 Obese (−): 10 Sickness (−): 5 Physical injury (−): 4 Multiple psychological burdens (−): 1 Likelihood of HIV infection (−): 1 Mortality rate (−): 1	
Parental education level	Oral health (+): 4	17
	Mental health issues (depression, etc.) (−): 6 Obese (−): 5 Illness (asthma) (−): 1 Physical injury (−): 1	
Family support	Life satisfaction (+): 2	5
	Depressive symptom (−): 2 Physical injury (−): 1	
The role of parenting style	Mental health (−): 1	1
Parents’ relationships	Mental health (+): 1	1

(+ means positive effect, − means negative effect).

**Table 9:** Analysis of social determinants of inequalities in health outcomes: social and community level.

Specific Influencing Factors	Health Behavior	No. of Studies
Community or neighborhood social capital	Oral health (+): 4 Physical health status (+): 1	9
	Obese (−): 2 Physical injury (−): 1 Substance use disorders (−): 1	
School social capital	Oral health (+): 6 Life satisfaction (+): 3	15
	Physical and mental health complaints (−): 6	
Peer social capital	Life satisfaction (+): 3	11
	Physical and mental health complaints (−): 7 Multiple psychological burdens (+): 1	

(+ means positive effect, − means negative effect).

**Table 10:** Analysis of social determinants of inequalities in health outcomes: general socioeconomic level.

Specific Influencing Factors	Health Behavior	No. of Studies
Socioeconomic deprivation	Perceived health (−): 2	3
	Adverse childhood experiences (+): 1	
Area of residence	Oral health (+): 5 Health-related quality of life (+): 1 Mental health status (+): 1	8
	Prevalence of heart disease (−): 1	
National income (e.g., GDP)	Suicide rate (−): 2 Physical and mental health complaints (−): 1 Obese (−): 1 Prevalence of heart disease (−): 1 Internalization of symptoms (−): 1	6
Social income inequality (e.g., Gini coefficient)	Nutrition and health status (−): 1	11
	Clinical depression (+): 3 Obese (+): 3 Physical and mental health complaints (+): 2 Suicide rate (+): 2	
Gender inequality	Physical injury (+): 1	1
Stratification of the national education system	Physical and mental health complaints (−): 1	1
Religious discrimination	Sleep health (−): 1	1
Social cohesion	Perceived health (+): 3	4
	Clinical depression (−): 1	
Social inclusion	School completion (+): 1	1
COVID-19	Mental health status (−): 2	3
	Overweight (+): 1	

(+ means positive effect, − means negative effect).

## Data Availability

No new data were created or analyzed in this study. Data sharing is not applicable to this article.
